# Cognitive-Motor Interference While Grasping, Lifting and Holding Objects

**DOI:** 10.1371/journal.pone.0080125

**Published:** 2013-11-07

**Authors:** Erwan Guillery, André Mouraux, Jean-Louis Thonnard

**Affiliations:** Institute of Neuroscience, Université catholique de Louvain, Brussels, Belgium; VU University Amsterdam, Netherlands

## Abstract

In daily life, object manipulation is usually performed concurrently to the execution of cognitive tasks. The aim of the present study was to determine which aspects of precision grip require cognitive resources using a motor-cognitive dual-task paradigm. Eighteen healthy participants took part in the experiment, which comprised two conditions. In the first condition, participants performed a motor task without any concomitant cognitive task. They were instructed to grip, lift and hold an apparatus incorporating strain gauges allowing a continuous measurement of the force perpendicular to each contact surface (grip force, GF) as well as the total tangential force applied on the object (load force, LF). In the second condition, participants performed the same motor task while concurrently performing a cognitive task consisting in a complex visual search combined with counting. In the dual-task condition, we found a significant increase in the duration of the preload phase (time between initial contact of the fingers with the apparatus and onset of the load force), as well as a significant increase of the grip force during the holding phase, indicating that the cognitive task interfered with the initial force scaling performed during the preload phase and the fine-tuning of grip force during the hold phase. These findings indicate that these aspects of precision grip require cognitive resources. In contrast, other aspects of the precision grip, such as the temporal coupling between grip and load forces, were not affected by the cognitive task, suggesting that they reflect more automatic processes. Taken together, our results suggest that assessing the dynamic and temporal parameters of precision grip in the context of a concurrent cognitive task may constitute a more ecological and better-suited tool to characterize motor dysfunction in patients.

## Introduction

In everyday life, object manipulation is among the most common tasks we perform. Irrespective of the final goal, it generally involves grasping, lifting and holding objects. Despite its apparent easiness, grasping and loading an object involves subtle interplay between predictive and feedback neural controls in order to generate a smooth vertical acceleration of the object [1–3]. Visual cues provide information about most of the mechanical properties of the object (size, shape, etc.) that is useful to predict the forces required for successful manipulation [4]. In addition, tactile input directly provides information about the mechanical interactions between our hands and objects (friction between skin and object, timing, magnitude and direction of fingertip forces) [1,5,6].

Purposeful objects manipulation requires the ability to adapt in function of individual goals and environmental constraints [7]. Therefore, object manipulation cannot be considered as a series of rote repetitions with each movement exactly like the last, as the environment and the purpose of the movement varies considerably. Instead, object manipulation is a complex task requiring integration of sensory, motor and cognitive systems [8,9]. Most importantly, object manipulation in daily life is usually performed concurrently with other cognitive tasks such as attending a conversation or recalling a shopping list.

Dual-task paradigms have been used for many years to make inferences regarding the nature of the processing resources recruited during the performance of various tasks [10,11]. By examining the impact of a primary task on performance of a secondary task, cognitive-motor dual-task paradigms have been used to explore the attentional demands of a motor task [12,13]. If an interaction is revealed, it can be assumed that both tasks compete for the same mental resources at some time during the execution of the two tasks [10]. Using such paradigms, previous studies have already shown that a concurrent cognitive task can interfere with the realization of upper limb movements [8,14–20]. Most of these studies reported that the early stages of the movement, i.e. the planning phase, can be affected by the cognitive task [8,15,16,19,21–23]. Depending of the nature of the cognitive task, some studies reported that the online control of movement during the execution phase is also affected [23,24] whereas other studies failed to demonstrate such an effect [14,15,19]. The fact that different aspects of movement may involve specific aspects of cognition is highlighted by the results of Spiegel et al. [24], suggesting that spatial working memory interferes more with the execution of a movement than verbal working memory.

Only a few studies have focused on the effect of a cognitive task on the dynamic of precision grip movement [17,20] and, to our knowledge, no studies have explored the effects of an unrelated cognitive task on grip-lift-hold coordination. The aim of the present study was to use a dual-task interference paradigm, to characterize how performing such a cognitive task interferes with object manipulation in healthy individuals. Specifically, we applied a dual-task paradigm to assess cognitive-motor interferences while grasping, lifting and holding an object between the thumb and index finger, i.e. precision grip. The cognitive task consisted in a complex visual search combined with counting. The object manipulated by the participants was instrumented with sensors allowing an online recording of the force perpendicular to each contact surface (grip force) as well as the tangential force applied on the object (load force). This allowed us to characterize precisely the involvement of cognitive resources for each of the different temporal and dynamic aspects of precision grip. We found that the grip force applied onto the object, as well as the delay separating the initial contact of the fingers with the object and the onset of the load force applied onto the object were increased in the dual-task condition, indicating that these two aspects of precision grip rely on cognitive processes. In contrast, other aspects of the precision grip – in particular the temporal coupling between grip and load forces – were not affected by the cognitive task. Taken together, our results suggest that assessment of dynamic and temporal parameters of the precision grip in dual-task paradigms could provide a more ecological and better-suited tool to characterize motor dysfunction in patients.

## Materials and Methods

### Participants

All participants provided written informed consent. The study was approved by the ethical committee of the Université catholique de Louvain, (B4032006615). Eighteen healthy naïve volunteers (undergraduate and graduate students of the university) took part in the experiment, (9 men and 9 women; mean age: 26 ± 3, range 22-29). None presented motor and/or cognitive deficits. All had normal or corrected to normal vision. According to the Edinburgh Oldfield Handedness Inventory [25], sixteen participants were right-handed and two were left-handed. Participants did not receive financial compensation for their participation.

### Apparatus

**Figure 1:**
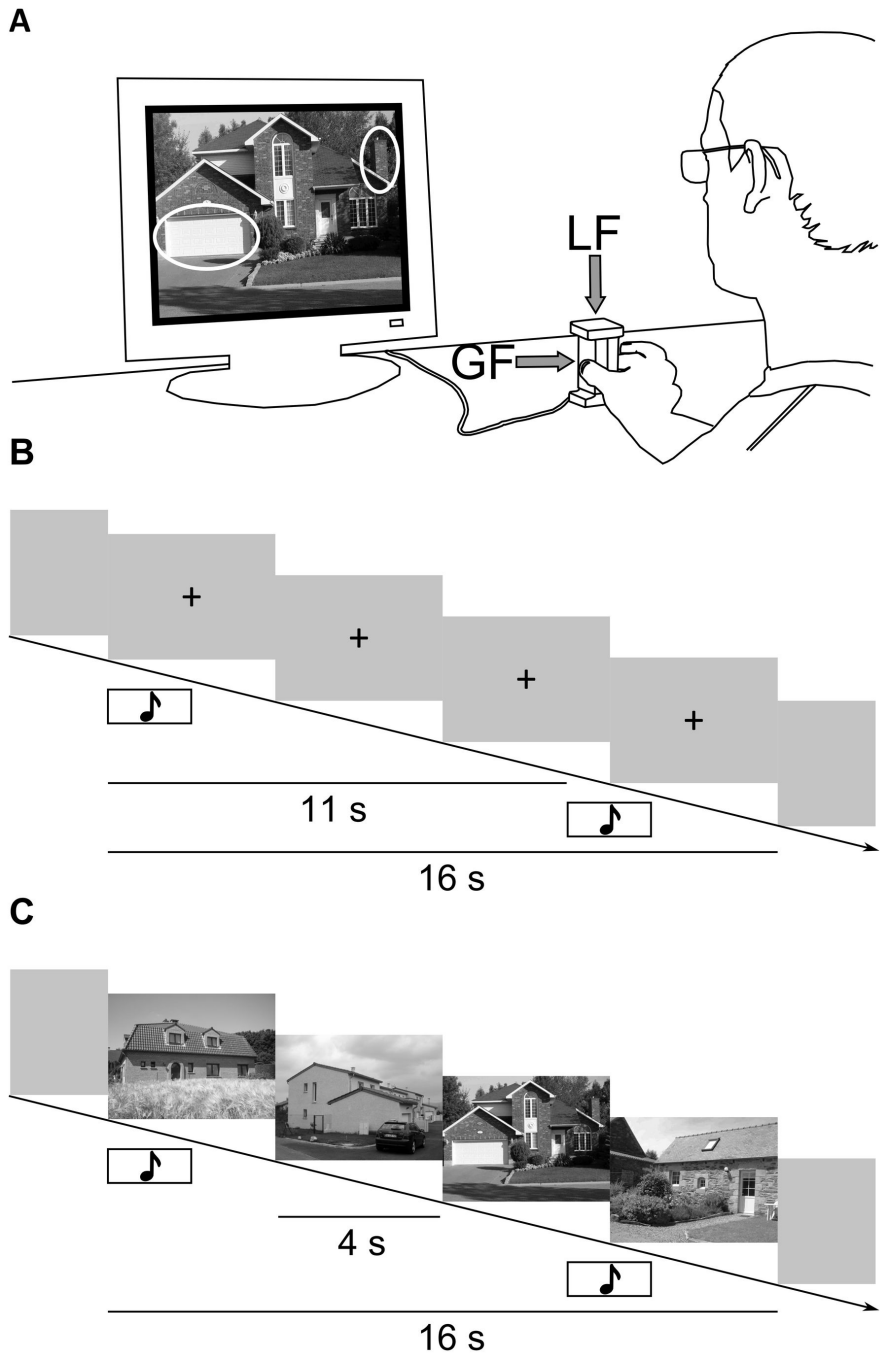
Experimental procedures. A. Participants were seated in front of a computer display and grasped the apparatus between the thumb and the index. The device was equipped with strain gauges measuring the grip force (GF) and load force (LF) developed during the experiment. B. In the “motor” condition (M), an auditory tone prompted the participant to grip, lift and maintain the apparatus approximately 5 cm above the table. After 11 s, a second auditory tone prompted the participant to put down the apparatus on the table and to reposition their hand at rest next to the apparatus. During the task, the participants fixated a cross displayed at the centre of the computer display. C. In the “motor + cognitive” condition (M+C), participants performed a visual search and counting task concomitant to the motor task. At the first tone, a colour photograph including a house was displayed on the computer screen. The photograph was changed every 4 s. A total of 4 pictures were shown in each trial. Participants were asked to count the number of pictures in which a chimney and a car, or a chimney and a garage could be identified. They reported their answer verbally at the end of each trial.

The apparatus used for these experiments was a 275 g, 108 x 56 x 38 mm (height, width, and depth) mechanical assembly (Figure 1) (fMRI-GLM, Arsalis, Belgium). The device was instrumented with full Wheatstone Bridges incorporating three strain gauges load sensors allowing to measure the force perpendicular to each contact surface (grip force*s*, GF left and GF right) as well as the tangential force applied on the object (load force, LF) [26]. It was calibrated up to a full scale of 30 N in each direction and demonstrated a maximum nonlinearity of 0.70 % for LF and 0.35 % for GF. The analogue signals were amplified, filtered with a Bessel 4-pole 150 Hz cut-off low-pass filter and sampled at 2000 Hz with a resolution of 16-bit. The resolution of the force measurements was 0.002 N for GF and 0.001 N for LF. The data was stored on a personal computer for offline analysis.

Visual and auditory stimuli were generated using Matlab 6.5 (The MathWorks Inc., USA) and the Cogent 2000 graphics toolbox (http://www.vislab.ucl.ac.uk/cogent_2000). Pictures and instructions were displayed on a 17 inch LCD monitor (AL1703sm, Acer Inc, USA) positioned approximately 1 m in front of the participant, using a 800x600 resolution and a 60 Hz refresh rate.

### Experimental Design

Participants sat comfortably in a chair in front of a desk supporting the apparatus and LCD monitor. They were instructed to keep their non-dominant hand at rest, and their dominant hand around the apparatus (the thumb and index fingertips were positioned approximately 1 cm from the center of the contact surfaces of the object) (Figure 1). Before the beginning of each experimental session, participants had to wash their hands to reduce interindividual variability in finger skin friction. Each task was explained carefully. The experiment comprised two conditions. In the first condition (M), participants performed a motor task without any concomitant cognitive task. In the second condition (M+C), participants concomitantly performed the same motor task and a cognitive task.

The motor task was a grip-lift movement [27]. An auditory tone (2 s, 1100 Hz) prompted the participants to grip, lift and maintain the apparatus approximately 5 cm above the table. A second auditory tone (2 s, 700 Hz), occurring 11 s after the onset of the first, prompted the participants to put down the apparatus on the table and to reposition their hand at rest around to the apparatus. During the motor task, the participants were asked to fixate a black cross positioned at the center of the screen (Figure 1). Five seconds separated the end of each trial from the beginning of the following trial. This ensured that participants had enough time to reposition their hand next to the apparatus.

The cognitive task consisted in a visual search and counting task. At the first tone, a colour photograph including a house was displayed onto the computer screen. The photograph was changed every 4 s. A total of 4 pictures were shown in each trial. Participants had to recall the number of pictures in which a chimney and a car, or a chimney and a garage could be identified. Participants reported their answer verbally at the end of each trial. The visual search task lasted 16 seconds to ensure it covered the entire duration of the motor task including the grip, lift, hold and release phases, and to avoid any additional interference by the subject’s verbal response to the cognitive task. Each picture was shown only once throughout the experimental session. Half of the presented pictures were targets (i.e. pictures showing a chimney and a car or a chimney and a garage). The order of the pictures was the same for each participant.

In both the M and the M+C conditions, the procedure was repeated ten times. Importantly, because participants were required to fixate the computer screen in both conditions, visual feedback related to the hand/object position was identical in the two conditions. Participants were given the opportunity to practice each condition (ten consecutive trials for each condition). The order of the conditions (M and M+C) was counterbalanced between participants.

Furthermore, for each participant, the skin-apparatus coefficient of static friction was measured three times: before the practice trials, before the recording trials and after the recording trials, in a series of eight lift-and-drop manoeuvres during which the participant lifted and held the instrument stationary, then gradually released the grip until the object slipped due to gravity [27].

### Data analysis

**Figure 2:**
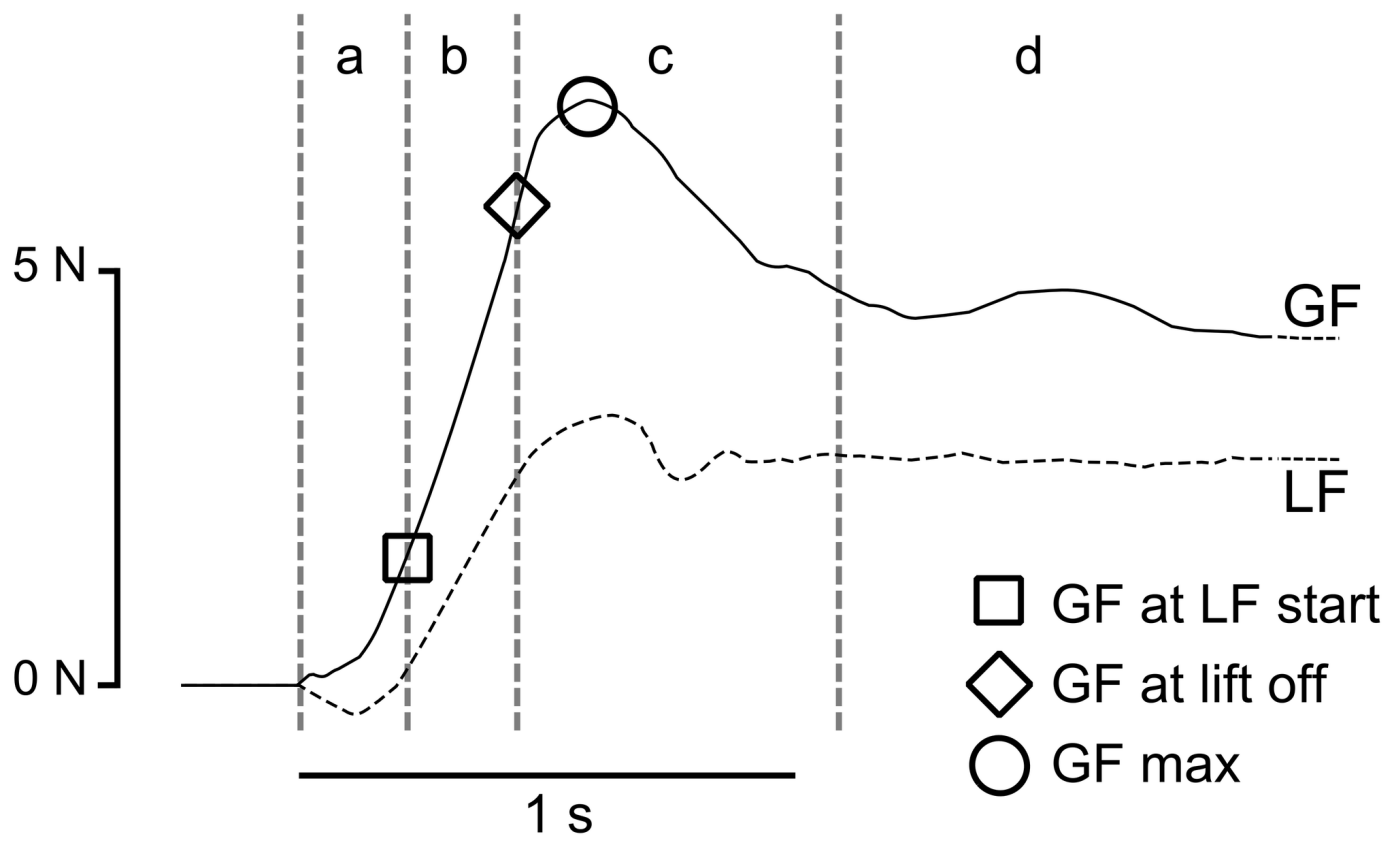
Time course of grip force (GF: continuous waveform) and load force (LF: dotted waveform) during the grip and hold task. Several measures were extracted from these waveforms. The preload phase (a) corresponds to the duration separating the first contact of one finger on the apparatus and the onset of a positive load force (LF). The load phase (b) corresponds to the time during which a parallel increase of GF and LF is observed. The lift phase (c) corresponds to the time during which the apparatus is raised and stabilized. The hold phase (d) corresponds to the time during which the object is maintained in a stable position. GF at LF start corresponds to the value of GF at the beginning of the load phase. GF at lift off corresponds to the value of GF when LF equals the weight of the apparatus, i.e. when the apparatus begins lift off. GF max correspond to the maximum values of GF .Values are in Newton (N) and second (s).

Forces were analysed using Matlab 7.5 (The MathWorks, Inc., USA). Signals were low-pass filtered using a Butterworth filter (cut-off: 15 Hz; slope: 2dB). Typical GF and LF traces are shown in Figure 2. These traces can be decomposed into distinct phases [1]: (a) the preload phase, defined as the time between initial contact of the fingers with the apparatus and onset of the load force, (b) the load phase during which both GF and LF increase up to the point where LF equals the weight of the apparatus (2.75 N), i.e. the point where the apparatus is lifted from the table, (c) the lift phase corresponding to the time interval before stabilization of the apparatus above the table and (d) the hold phase corresponding to the time interval during which the apparatus remains stable above the table. In addition to the duration of these phases, the following parameters were compared across conditions: the GF at onset of the load force (LF > 0.1 N) and at lift off which provides information about the grip force applied at the very early stage of the movement, i.e. at the beginning and the end of the load phase, the maximum GF; and the mean and standard deviation of GF during the hold phase.

For each lift-and-drop manoeuvres, the static coefficient of friction (CF) was estimated as half the LF/GF ratio at slip onset [1]:

CF=LFat slip/2GFat slip

This was used to compute the safety margin (SM) providing information on the excess of grip force applied onto the object. It corresponds to the ratio of two forces and therefore has no unit.

SM=(GFhold−GFat slip)/GFhold

with

GFat slip=LFhold/2CF

Finally, the temporal coupling of load and grip force was assessed using a cross correlation analysis [28] between the first derivative of LF (dLF/dt) and the first derivative of GF (dGF/dt) in the time-interval separating the first contact of one finger on the apparatus and GF max. This measure involves progressively sliding one waveform past the other and computing the correlation coefficient between the time-shifted signals. The cross-correlation function peaks at time lags where the two waveforms are best aligned. The analysis thus provides two values: (a) the time lag at maximum correlation which provides an estimate of the time lag between the two signals and (b) the maximum coefficient of correlation which provides an estimate of the similarity between the two time courses. To assess the effect of the cognitive task on the asynchrony between GF and LF, we computed the absolute value of time lags.

### Statistical analyses

The measures obtained in each of the two conditions (M vs. M+C) were compared using paired t-tests when they followed a normal distribution and using a Wilcoxon signed rank test when they did not (Sigma Stat 3.5, SPSS Inc., USA). Normality was tested using the Kolmogorov-Smirnov test. To address the problem of multiple comparisons, the significance level was corrected using a False Discovery Rate (FDR) procedure [29]. A One-Way Repeated Measure Analysis of Variance on ranks was performed to test values of CF obtained at the different time points (before the practice trials, before the recording trials and after the recording trials).

## Results

The cognitive task was correctly performed by each participant (89 ± 9% of correct answers; mean ± standard deviation).

Table 1 presents the group-level mean and standard deviation of each measured dynamical and temporal parameters of the grip-lift task performed in the M and M+C conditions, as well as the results of the comparison between the two conditions.

**Table 1:** Mean and standard deviation (SD) of Temporal and Dynamic parameters of the M and M+C conditions.

**Temporal Parameters**	**M**	**M + C**	**M vs M + C**		
	Mean ( SD )	Mean ( SD )	*t*-test	*p*-value	*Sig.*
Preload Phase (ms)	230 (91)	285 (104)	2.88	0.010	*
Load Phase (ms)	214 (51)	209 (48)	0.40	0.691	
Lift Phase (ms)	688 (105)	661 (75)	1.54	0.141	
Cross-correlation Coefficient	0.92 (0.03)	0.91 (0.03)	1.64	0.120	
Absolute Time-Lag (ms)	26 (17)	17 (11)	0.81^1^	0.442	
**Dynamical Parameters**	**M**	**M + C**	**M vs M + C**		
	Mean ( SD )	Mean ( SD )	*t*-test	*p*-value	
GF at LF start (N)	1.28 (0.76)	1.39 (0.74)	0.15^1^	0.899	
GF at lift off (N)	5.72 (0.82)	6.28 (1.05)	2.30	0.035	
GF max (N)	6.57 (0.83)	7.18 (1.01)	3.09^1^	0.007	*
GF hold (N)	4.56 (0.56)	5.07 (0.61)	2.97^1^	0.009	*
Standard deviation of GF hold (N)	0.36 (0.12)	0.37 (0.13)	0.31	0.759	
Safety Margin	0.53 (0.06)	0.58 (0.05)	3.47	0.003	*

Significant results (*p* < .05; corrected using the Benjamini & Hochberg False Discovery Rate procedure [29]), are highlighted with an asterisk. “1” indicates when Signed Rank test was performed.

### Temporal parameters

The preload phase was significantly lengthened in the M+C condition (*t*(17)=2.88; *p*=0.010). On average, it was 55 ± 80 ms longer in the M+C condition as compared to the M condition. In contrast, the concurrent cognitive task did not interfere with the duration of the load phase (*t*(17)=0.40; *p*=0.691) and the duration of the lift phase (*t*(17)=1.54; *p*=0.141).

The cross-correlation function did not reveal any change in the temporal coupling between GF and LF The average absolute time lag between GF and LF was 26 ± 17 ms in M condition and 17 ± 11 ms in the M+C condition This difference was not significant (*z*=0.81; *p*=0.442). The average of maximum cross-correlation coefficients was 0.92 ± 0.03 in the M condition and and 0.91 ± 0.03 in the M+C condition. This difference was not significant (*t*(17)=1.64; *p*=0.141).

### Dynamical parameters

GF max was significantly increased in the M+C condition as compared to the M condition (*t*(17)=3.09; *p*=0.007). On average, GF max was increased by +0.61 ± 0.84 N in the M+C condition A significant increase of GF was also observed during the hold phase (+0.51 ± 0.74 N; *t*(17)=2.97; *p*=0.009). GF was increased at lift off (+0.55 ± 1.02 N; *t*(17)=2.30; *p*=0.035), but this difference was not significant after FDR correction for multiple comparisons. In contrast, GF at the beginning of the load phase (GF at LF start) and the standard deviation of GF during the hold phase were not significantly different in the M and M+C conditions (*z*=0.15; *p*=0.899 and *t*(17)=0.31; *p*=0.759).

Values of CF did not follow a normal distribution; a Kruskal-Wallis one-way repeated measure analysis of variance on ranks was performed. The differences among the values of CF were not significant (*h*(2)=0.71; *p*=0.703). Thus we assumed that participants showed a similar CF in all conditions.

The SM, which depended on the GF exerted during the hold phase, was significantly different in the M and M+C conditions (*t*(17)=3.47; *p*=0.003). On average, participants increased their safety margin from 0.53 ± 0.06 in the M condition to 0.58 ± 0.05 in the M+C condition.

## Discussion

The objective of this study was to investigate cognitive-motor interference by assessing the interactions between a precision handgrip motor task and an unrelated cognitive task. This allowed us to examine the involvement of high-level cognitive resources in the performance of a common manual behaviour. In the dual-task condition, we found a significant increase in the duration of the preload phase, as well as a significant increase of the grip force during the hold phase. In contrast, the other aspects of the precision grip, such as the temporal coupling between grip and load forces were not affected by the cognitive task.

The preload phase was significantly increased when performing the concurrent cognitive task. The preload phase is critical to encode somatosensory input generated by the initial contact with the object. It has been shown by Westling and Johansson [30] that, during the preload phase, fast adapting and slowly adapting type 1 mechanoreceptors encode afferent information that is decisive for the release of the motor commands accounting for the load phase. It seems obvious that, during the preload phase, the central nervous system needs information indicating a reliable contact before releasing the muscle commands leading to the load phase. Previous studies have shown that the duration of the preload phase is increased in very young children [31], in older adults [32], in children and adults with hemiplegia [28,33] and in healthy adults following repetitive transcranial magnetic stimulation of the primary somatosensory cortex [34]. In most of these studies, it seems likely that the increased duration of the preload phase results from an altered cortical processing of the sensory signals generated by the contact of the fingers with the object and/or integration of that information into the movement plan. Our finding that the duration of the preload phase is increased in the dual-task condition implies that at least some of these processes involve cognitive resources and, hence, correspond to controlled processes rather than purely automatic processes. One possibility is that the increased duration of the preload phase in the dual-task condition is due to the fact that cortical processing of the somatosensory input generated by the contact of the finger with the object is modulated by the focus of selective attention, or by the availability of working memory resources. This view is also supported by the results of previous studies showing that movement planning requires working memory resources and can interfere with concurrent cognitive processing [8,15,16,19,21–23].

Another explanation to consider is that, in the dual-task condition, the increased duration of the preload phase was due to the fact that the participants already increase grip force during this phase of the movement. However this hypothesis can be rejected as the grip force at the beginning of the load phase (GF at LF start) was not increased in the M+C condition.

The maximum grip force (GF max) and the grip force during the holding phase (GF hold) were the two other factors significantly increased during the dual-task condition. One possible explanation to the increased grip force is that participants anticipated the interference of the concurrent cognitive task by increasing the safety margin and, thereby, reducing the risk of letting the object slip because of less optimal planning and/or fine-tuning of the grip force.

During the hold phase, the grip force is thought to be adjusted through reactive mechanisms relying on tactile feedback [1]. In a highly economical fashion, the grip force would be maintained just above the force under which the object would slip. This is supported by the fact that, when peripheral sensory information is impaired, the grip force is increased because the adjustment of grip force is no longer optimal, as shown in healthy participants with local anaesthesia of the index and thumb [35], in older adults [32], under mental stress in microgravity and hypergravity environments environments [36,37] and in patients with peripheral nerve lesions [38,39]. In the present study, we observed a similar increase of grip force in the dual-task condition, thus suggesting that participants adopted a similar behaviour to compensate the fact that the cognitive task interfered with the fine adjustment of grip force during the hold phase. Few studies have examined the impact of attentional disorders on the performance of a precision grip lift task. Pereira et al. [40,41] showed that children with attention-deficit-hyperactivity disorder (ADHD), and children with deficits in attention motor control and perception (DAMP), have deficient control of fingertip forces during precision grip lift movement. They attributed this impairment to a general deficit of working memory function. Our findings provide further evidence that the fine-tuning of grip force during the hold phase involves cortical processes that are dependent on the cognitive resources required to perform an unrelated cognitive task involving selective attention and/or working memory function [24].

One possible explanation to the increase in grip force observed during the dual-task condition could be that this increase compensated a reduction in the coefficient of friction between the fingertips and the manipulated object. Indeed, the difficulty of the dual-task could have induced an autonomic response leading to a change in skin fingertip moistness, a factor known to crucially determine the coefficient of friction [42,43]. However, this was not the case, as we did not observe any significant difference in the coefficient of friction across trials. 

Contrasting with the effect of the cognitive task on the preload phase and grip force, there was no significant difference in the temporal coupling of grip and load forces, as assessed by the cross-correlation between the two measures [44]. This suggests that this coupling may rely on automatic processes that are less dependent or do not involve cognitive resources.

Although participants were asked to focus their gaze on the computer screen in both conditions, actual eye movements were not controlled. As fixation can affect reaching and grasping behaviour, this limitation of the present study could be addressed in future studies, using eye tracking methods to monitor eye gaze in the different conditions.

Altogether our findings show that mental ressources are required for both the planning and the online control of upper-limb movement [23,24]. Using a similar dual-task paradigm, future studies should examine the influence of a cognitive task on the different aspects of precision grip in patients presenting with a peripheral or central lesion of the nervous system affecting the transmission and processing of sensory input and motor output (e.g. sensory and motor impairment following stroke and/or peripheral neuropathy). Indeed, it could well be that following such a lesion, precision grip is even more dependent on cognitive resources. This is of particular importance as previous studies have largely failed to demonstrate decisive changes in precision grip following specific rehabilitation procedures, although patients often report a subjective improvement of their ability to conduct daily-life activities involving the manipulation of objects [26]. Therefore, it would be of interest to examine whether these rehabilitation procedures reduce the impact of a concurrent cognitive task on precision grip, which would suggest that the rehabilitation reduced the amount of cognitive resources required for object manipulation [20,45]. In other words, studying precision grip in the context of a concurrent cognitive task may constitute a more ecological way to study motor function, which could be especially useful for the clinical evaluation of patients and the assessment of treatment efficacy.
